# Discrimination and Sleep in Chinese American Older Adults

**DOI:** 10.1093/geroni/igab046.3588

**Published:** 2021-12-17

**Authors:** Alexander Dong, Stephanie Bergren, Qun Le, Lisa Lanza, Dana K Dychtwald

**Affiliations:** 1 Rutgers University, New Brunswick, New Jersey, United States; 2 Rutgers University, Rutgers Institute for Health, New Jersey, United States

## Abstract

Since the start of COVID-19, reports of discrimination in the US against Asian Americans have increased approximately 150%. Prior research has demonstrated that victims of discrimination are more likely to experience physiological health concerns, possibly linked to sleep. The objective of this study was to determine if there was a relationship between disordered sleep and discrimination among Chinese older adults using data collected from the Population Study of ChINese Elderly (N=3124, 59% female). To assess, the experience of discrimination in nine settings (school, hiring, work, housing, medical, service, finance, public, and authority) and four indicators of sleep quality (duration, trouble falling asleep, insomnia, and self-reported sleep quality) were evaluated using logistic- and multinomial logistic regression. With an average age of 75 years, discrimination was experienced by 7.2% of participants. Experiencing any discrimination was associated with lower odds of longer sleep durations (>8 hours) compared to those sleeping 6-8 hours. Experiences of discrimination in housing (OR: 5.51 (95%CI:1.08-27.98)) and with authority figures (OR: 6.02 (95%CI:1.16-31.31)) were significantly associated with shorter sleep durations (<6 hours), compared to those sleeping 6-8 hours. Those who experienced discrimination in a school setting were less likely to have trouble falling asleep (OR: 0.28 (95%CI:0.09-0.88)), while discrimination in medical settings were more likely to experience insomnia (OR: 2.29 (95%CI:1.13-4.63)). All other relationships between discrimination and sleep measures were non-significant. Given mixed evidence and the increased relevancy of discrimination against Asian Americans, further research on how discrimination may impact health outcomes and sleep quality is warranted.

